# Efficacy of acupuncture in the prevention and treatment of chemotherapy-induced nausea and vomiting in patients with advanced cancer: a multi-center, single-blind, randomized, sham-controlled clinical research

**DOI:** 10.1186/s13020-020-00333-x

**Published:** 2020-06-03

**Authors:** Qi-Wei Li, Ming-Wei Yu, Xiao-Min Wang, Guo-Wang Yang, Huan Wang, Chen-Xi Zhang, Na Xue, Wei-Ru Xu, Yi Zhang, Pei-Yu Cheng, Lin Yang, Qi Fu, Zhong Yang

**Affiliations:** 1 Beijing Hospital of Traditional Chinese Medicine Affiliated with Capital Medical University, No 23, Back Road of Art Gallery, Dong Cheng District, Beijing, 100010 China; 2 Beijing University of Chinese Medicine, No. 11, North 3rd Ring East Road, Chaoyang District, Beijing, 100029 China

**Keywords:** Acupuncture, Chemotherapy-induced nausea and vomiting, Cancer

## Abstract

**Background:**

Chemotherapy-induced nausea and vomiting (CINV) is a common and distressing side effect. We conducted this clinical trial to compare the effectiveness of true acupuncture vs. sham acupuncture in controlling chemotherapy-induced nausea and vomiting (CINV) among patients with advanced cancer.

**Methods:**

A total of 134 participants were randomly allocated into true acupuncture (TA) (n = 68) and sham acupuncture (SA) (n = 66) groups. Participants in both groups received acupuncture session twice on the first day of chemotherapy, and once consecutively on the following 4 days. The primary outcome was using the Common Terminology Criteria for Adverse Events (CTCAE) to assess CINV. The secondary outcome measures were the Eastern Cooperative Oncology Group score (ECOG), Simplified Nutritional Appetite Questionnaire (SNAQ), and Hospital Anxiety and Depression scale (HADS).

**Results:**

Compared to the SA group, the TA group didn’t show significant improvement in complete response rates of chemotherapy-induced nausea and vomiting (all P > 0.05). However, the TA group could modestly reduce the severity of nausea (from day-3 to day-21, P < 0.05) or vomiting (from day-4 to day-21, P < 0.05), which is notably superior to the control group. Besides, TA promoted the nutritional status of patients with a significantly higher score comparing to the SA group on day 14 (21.82 vs.20.12, P = 0.003) and day 21 (22.39 vs. 20.43, P = 0.001). No apparent differences were found in anxiety and depression assessment between these groups. Participants in both groups were well tolerant of acupuncture therapy. There was no adverse event occurs in our study.

**Conclusion:**

Acupuncture as an adjunctive approach could alleviate the severity of chemotherapy-induced nausea and vomiting compared to the sham control, even though the effect of acupuncture in preventing CINV occurring is relatively modest.

## Background

Chemotherapy-induced nausea and vomiting (CINV) is one of the most common and debilitating side effects which exerts a detrimental influence on the quality of life (QOL) of cancer patients and reduces their compliance towards conventional therapies. CINV typically presents in two phases, the acute phase and the delayed phase [1]. Acute CINV occurs within 1–2 h of chemotherapy administration and can last for up to 24 h, while delayed CINV phase presents more than 24 h after chemotherapy until 120 h.

Nowadays, the development of prophylaxis for chemotherapy-induced emesis has dramatically relieved chemotherapy-induced emesis. Patients receiving highly emetogenic chemotherapy (HEC) are recommended with a four drug combination of a neurokinin 1 (NK_1_) receptor antagonist, a serotonin (5-HT_3_) receptor antagonist, dexamethasone, and olanzapine by the ASCO guideline [2]. A phase 3 trial reported this combination therapy achieved a complete response rate of 79% in the patients who were receiving the first circle of cisplatin-based chemotherapy [3]. However, this result was based on only a single circle of chemotherapy with cisplatin. Additionally, patients undergoing chemotherapy with anthracycline and cyclophosphamide had a more inadequate control of CINV in the acute phase than those who treated with cisplatin [4]. It could be speculated that the complete response rate of CINV by the standard antiemetic medications in clinical practice is lower than what is reported in the literature. Despite the advance in antiemetic therapy, practice patterns of antiemetic use revealed low adherence to those guidelines [5] due to unbalanced medical development, financial burdens, provider barriers, and patient factors in different countries and areas [6, 7]. Although emesis could be prevented well in most cases, nausea mainly occurring in delay-phase remains a significant challenge for cancer patients [4, 8, 9].

Efforts should be made to explore the economic and practical approaches for better symptom control of CINV. Integrative therapies, including acupressure and electroacupuncture, are recommended for clinical application with fewer side effects and high feasibility by ASCO guideline [10]. Acupuncture, as a component of traditional medicine systems, has been practiced in Asia for thousands of years and been well accepted worldwide. By stimulation on specific acupoints beneath the skin, acupuncture accommodates the flow of energy called *qi* throughout the body [11] and removes the blockages in meridians helping the body reestablishing homeostasis. Traditional Chinese acupuncture requires exerting manipulation on needles to present a sensation of *de qi* (a soreness, fullness, heaviness, or local area distension [12]).

It is reported by researchers from different countries with the optimized outcomes of acupuncture in controlling chemotherapy-induced emesis [12, 13]. Acupuncture could be considered as a supplementary therapy for relieving symptoms of CINV. Nevertheless, owing to the limitation of research quality, although acupuncture could be an appropriate adjunctive treatment for CINV, it still needs evidence of high-quality clinical researches according to a systematic review [14]. In our study, we performed a multi-center, single-blind, randomized, controlled trial to investigate the efficacy and safety of acupuncture in the prevention and treatment of chemotherapy-induced nausea and vomiting in patients with advanced cancer.

## Methods

This study was a multi-center, single-blind, randomized, sham-controlled clinical trial designed to investigate the effect of acupuncture versus sham acupuncture on CINV symptoms in patients with advanced cancer. All research procedures were approved by the Research Ethical Committee of Beijing Hospital of Traditional Chinese Medicine Affiliated to Capital Medical University (ref: 2014BL-067). All patients included in this study provided written, informed consent. All investigations were conducted in accordance with the Consolidated Standards of Reporting Trials statement [15] and the 2010 Standards for Reporting Interventions in Clinical Trials of Acupuncture [16]. The study protocol was registered on ISRCTN and Clinical-Trials.gov (ISRCTN13287728; Clinical-Trials.gov: NCT02369107). This trial protocol is attached as Additional file 1.

Participants were eligible for inclusion if they were diagnosed with lung cancer, breast cancer, or gynecological cancer and scheduled to receive cisplatin, anthracycline, or taxane-based chemotherapy regimens. Patients were required to be aged 18 years or older and aged 75 years or younger. The Eastern Cooperative Oncology Group performance status was 0–2. The expected lifetime was longer than 6 months. On the other hand, patients were excluded from joining this trial under the following conditions: severe cardiac arrhythmia, serious hepatorenal abnormal function, immune system, or hematopoietic system diseases, pregnant or lactating women. Patients with intractable vomiting caused by malignant brain metastases, intracranial hypertension, digestive tract obstruction, severe liver or renal dysfunction, brain tumors, cerebrovascular diseases, or other reasons couldn’t take part in the research. Acupuncture is a minimally invasive therapy. Thus, patients diagnosed with coagulopathy, thrombocytopenia, or other bleeding disorders were not allowed to join. It was not suitable in our study in patients with lymphedema in selected treating areas, fear of acupuncture, or allergy to needles. Other exclusion standards included that diagnosis with depression, anxiety disorders, psychosis, sepsis, or bacteremia.

Randomization was done using permuted block randomization with a secure computer system. Randomly allocated information was sealed in opaque envelopes and delivered to each center. Random allocation was performed after eligible participants who consented in written form and complete baseline assessments. Statisticians generated the allocation sequence. Primary investigators who were responsible for enrollment, and acupuncturist who conducted treatment were aware of the allocation. Patients were not informed of the group allocation. Investigators in charge of participant interviews and stuff for statistical analysis were also blind to the assignment. Blinding will be applied to the outcome assessment.

### Study interventions

All participants received chemotherapy regimens containing cisplatin, anthracycline, or taxane. Chemotherapy regimens were applied in accordance with the NCCN guidelines [17]. All patients were given pre-medications, including dexamethasone 20 mg and ondansetron (Ondansetron Hydrochloride Injection, Ningbo Team Pharm Co. Ltd, Ningbo, China) 8 mg intravenously as a foundation antiemetic regimen.

The trial lasted for 21 days, including 5-day treatment (from the chemotherapy initiation) and 16-day follow-up periods. All the acupuncturists who participated in this trial were major in acupuncture for more than 3 years with acupuncture licenses (Chinese medicine practitioner license) and had undergone rigorous training in conducting this trial.

Patients in this trial were randomized in a 1:1 ratio into a TA or SA group receiving the following acupuncture treatment.

#### Treatment A

Manual acupuncture was done at RN12 (Zhongwan), LR13 (Zhangmen, bilaterally), RN6 (Qihai), ST25 (Tianshu, bilaterally), PC6 (Neiguan, bilaterally), ST36 (Zusanli, bilaterally) (in Additional file 2: Table S1). All these acupuncture points excepting ST36 were stimulated manually every 10 min. A battery-operated SDZ-II Electronic Acupuncture Treatment Instrument (Hua Tuo medical instruments Co., Ltd. Suzhou Jiangsu, China) was connected to the needle inserted in ST36 and a skin electrode 5 cm from ST36 lateral on both sides. Electrical stimulation continued for 30 min at alternating frequencies of 2/100 Hz within the max-tolerance intensity of each patient.

#### Treatment B

Sham acupoints were selected for patients in this control group. To maintaining the same quantity of stimulus uniform in two groups, patients in the control group use the same kind, size, and number of needles for the control group as for the TA group. The stimulation points do not belong to traditional Chinese medicine meridians.

Participants who received TA or SA treatment twice on the day of chemotherapy initiation, and once on the consecutive 4 days. Each acupuncture session took approximately 30 min. There are ten needles in each session for both groups. Sterile needles for single-use (Ande, made in Guizhou, China) are used in this study. 25-gauge (0.25 mm in diameter), 40 mm-long needles are used for both groups at limb and abdomen. Needles in the TA group will be inserted 10–35 mm in depth and manually manipulated to produce the peculiar sensation known as De Qi, adding electro-acupuncture stimulation. During the TA session, acupuncturists will add manipulation to the needles every other 10 min. While in the control group, needles will be inserted about 1–2 mm in-depth with no manipulation. Participants in both groups will receive ondansetron intravenously as a foundation antiemetic regimen.

The sham acupoints used in the control group.

The stimulation positions do not belong to traditional Chinese medicine meridians. The sham acupuncture points are chosen from three different areas on the body (the abdomen, the Inside of the upper limb, the lateral lower leg), which do not correspond to recognized acupuncture points and are deemed to have no therapeutic value.

Additional doses of ondansetron 4 mg would be given if patients experienced severe nausea or vomiting.

### Outcome measures

#### Primary outcome measure

The primary outcome was evaluated by The Common Terminology Criteria for Adverse Events (CTCAE 4.0), established by the US National Cancer Institute (NCI), which is a set of criteria for the standardized classification of adverse effects of drugs used in cancer therapy. It includes standards to assess nausea and vomiting. It was used at baseline, day 1, day 2, day 3, day 7 (± 1), day 10 (± 1), day 14 (± 1) and day 21 (± 1) of the chemotherapy period. Patients would fill in questionnaires to record the occurring frequency and extent of CINV during the treatment period. Vomiting was defined as a projection of gastric contents and an attempt to vomit without gastric contents out from the mouth. Nausea was established as an unpleasant feeling associated with the urge to vomit. Complete control of nausea or vomiting was defined as no nausea or vomiting.

#### Secondary outcome measure

The secondary outcomes include the following contents. All patients were evaluated by The Eastern Cooperative Oncology Group (ECOG) [18] score, and the scores should range from 0 to 2. Hospital Anxiety and Depression scale (HADS) [19] was used to estimate the degree of anxiety and depression of patients. The HADS includes fourteen items, seven of which related to anxiety and seven related to depression. Simplified Nutritional Appetite Questionnaire (SNAQ) was applied for self-assessment of nutritional conditions [20].

ECOG and SNAQ were assessed at baseline, day 3, day 7 (± 1), day 14 (± 1), day 21 (± 1) of the observation period. HADS were evaluated at baseline, day 3, and day 7 (± 1).

### Statistical analysis

Sample size calculations were based on pilot research, in which the complete response rate of nausea and vomiting was 32% and 59% [21], respectively. The true acupuncture group was anticipated to raise the complete response rate by 25%. To detect a significant difference between these two groups with a power of 80% (α = 0.05, two-sided), factoring in a 20% withdrawal rate, the total sample size required was 134 participants with a 1:1 ratio in two groups. Randomization sequence for allocation was generated by SAS version 9.1.3 software (SAS Institute Inc., Cary, NC, USA).

Statistical analysis was performed with SPSS22.0. The baseline characteristics of the two groups were analyzed by t-tests. The categorical data such as the complete response rate of nausea/vomiting, adverse events were analyzed with Mcnemar Chi square test. If the average intensity of nausea and vomiting, the score of HADS and SNAQ followed a normal distribution, an independent *t* test would be used. Otherwise, the data would be analyzed with the Wilcoxon rank-sum test. A P-value of less than 0.05 was considered as statistically significant.

## Results

Between March 2015 to July 2017, 158 eligible patients were enrolled from Beijing Shijitan Hospital, Beijing Friendship Hospital, Beijing Hospital of Traditional Chinese Medicine, and Guang’ Anmen Hospital. Guang’ Anmen Hospital was added as the fourth research center because the allocation progress was slower than we estimated.

**Fig. 1:**
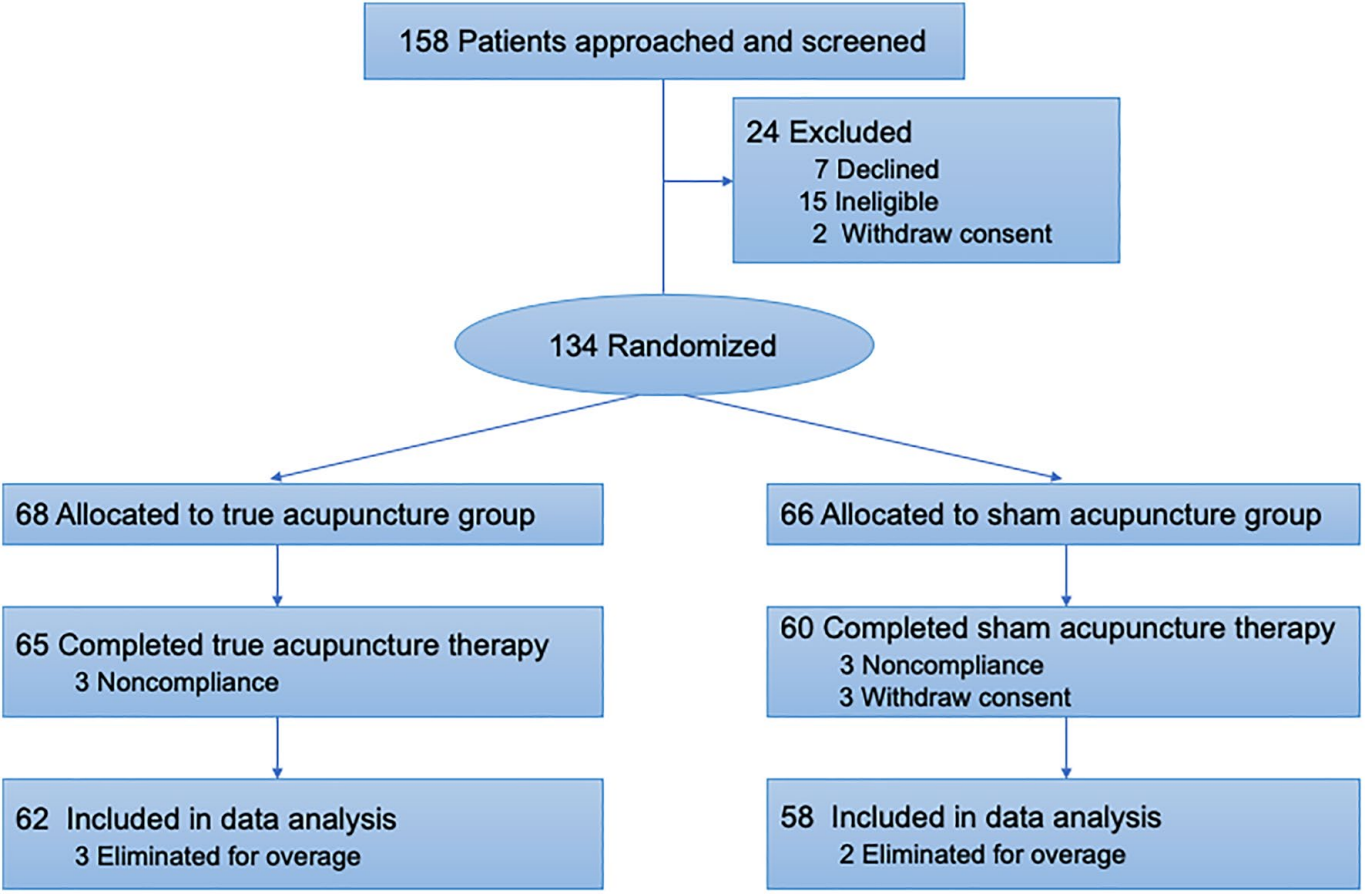
Flow of Randomized Patients for the Effect of True Acuounture vs Sham Acupuncture on CINV study

In the 158 patients, 15 patients failed to meet the inclusion criteria, while seven declined to participate in this research. There were 136 patients joined in the study, while two patients withdrawn their signed informed consent before the assignment due to the changing of minds. Finally, 134 participants were randomly assigned into the true acupuncture group (n = 68) and the sham acupuncture group (n = 66). In the follow-up phase, nine patients (6.6%) withdrew from this trial (3 in the true acupuncture group and 6 in the sham acupuncture group). As for the reason of drop-out, six patients transferred to another hospital for a therapeutic reason, and three patients revoked the consent forms for personal willingness. In the process of data cleaning, 5 cases were eliminated for failing to meet the inclusion criteria (over the up-age limit). Finally, 120 patients (including 62 in the true acupuncture group and 58 in the sham acupuncture group) were included in data analysis (Fig. 1). Patient baseline characteristics are shown in Table 1.

**Table 1:** Baseline Characteristics for Patients in the True Acupuncture and Sham Acupuncture Groups

	No. (%)		*P* value
	True acupuncture group (n = 68)	Sham aupuncture group (n = 66)	
***Age, median (95% CI)***	60 (56.84 ~ 60.48)	58 (54.99 ~ 60.04)	0.653
***Gender***			0.725
Male	14 (20.6)	12 (18.2)	
Female	54 (79.4)	54 (81.8)	
***Study site***			–
Beijing hospital of traditional Chinese medicine	22 (16.4)	22 (16.4)	
Guang’ anmen hospital	9 (6.7)	9 (6.7)	
Beijing friendship hospital	19 (14.2)	16 (11.9)	
Beijing shijitan hospital	18 (13.4)	19 (14.2)	
***Tumor pathological type***			0.830
Breast cancer	37 (54.4)	35 (53.0)	
Ovarian malignant carcinoma	8 (11.8)	8 (12.1)	
Cervical carcinoma	1 (1.5)	0 (0.0)	
Endometrial carcinoma	2 (2.9)	2 (3.0)	
Lung cancer	20 (29.4)	21 (31.8)	
***Cancer stage***			0.301
I	12 (17.6)	17 (25.8)	
II	18 (26.5)	16 (24.2)	
III	12 (17.6)	11 (16.7)	
IV	26 (38.2)	21 (31.8)	
***Prior chemotherapy***	33 (48.5)	30 (45.5)	0.721
***Chemotherapy regimen contains***			
Cis-platinum	28 (41.1)	17 (25.8)	0.059
Anthracycline	14 (20.6)	19 (28.8)	0.271
Taxane	32 (47.0)	38 (57.6)	0.223

*CI* confidence interval

*P values are based on independent-samples t-tests comparing differences between groups in age and whether or not before chemotherapy, *P* values in the study site, pathological tumor type, cancer stage, and chemotherapy regimen are based on Wilcoxon signed-rank test, and other *P* values are based on χ^2^ tests

## Primary outcomes

A comparison of the CINV degree in complete response rate (CRR) between two groups is exhibited in Table 2a, b). Similar emetic CRR in the 21-day observation. Although since the day 3 the CRR for nausea and vomiting in the true acupuncture group was slightly higher than that of sham acupuncture group, there is no statistical difference between two groups excepting for the CRR of nausea on the last day of chemotherapy circle (83.9% to 67.2%, respectively, *P *= 0.033).

**Table 2:** Observed and Fitted Group Assessment of CINV in Each Group

***a. CTCAE assessment of CINV***			
	Mean (SD) True acupuncture group (n = 62)	Sham acupuncture group (n = 58)	*P* value
***Nausea***			
Baseline	0.56 (0.692)	0.40 (0.528)	0.244
Day 1	0.82 (0.779)	0.67 (0.636)	0.326
Day 2	0.92 (0.685)	0.91 (0.779)	0.921
Day 3	0.87 (0.735)	1.02 (0.761)	0.288
Day 7 (± 1)	0.60 (0.689)	0.71 (0.622)	0.257
Day 10 (± 1)	0.37 (0.579)	0.48 (0.655)	0.355
Day 14 (± 1)	0.31 (0.531)	0.40 (0.591)	0.388
Day 21 (± 1)	0.18 (0.426)	0.39 (0.620)	0.041*
***Vomiting***			
Baseline	0.29 (0.555)	0.16 (0.451)	0.096
Day 1	0.52 (0.695)	0.30 (0.533)	0.078
Day 2	0.50 (0.741)	0.60 (0.771)	0.403
Day 3	0.50 (0.647)	0.57 (0.704)	0.656
Day 7 (± 1)	0.29 (0.584)	0.34 (0.579)	0.394
Day 10 (± 1)	0.19 (0.438)	0.36 (0.693)	0.177
Day 14 (± 1)	0.21 (0.484)	0.31 (0.681)	0.499
Day 21 (± 1)	0.10 (0.349)	0.23 (0.598)	0.183

***b. Completed control rate of CINV***			
	No. (%) True acupuncture group (n = 62)	Sham acupuncture group (n = 58)	*P* value
***Nausea***			
Baseline	34 (54.8)	36 (62.1)	0.422
Day 1	25 (40.3)	24 (41.4)	0.906
Day 2	17 (27.4)	20 (34.5)	0.402
Day 3	21 (33.9)	16 (27.6)	0.456
Day 7 (± 1)	32 (51.6)	22 (37.9)	0.132
Day 10 (± 1)	42 (67.7)	35 (60.3)	0.398
Day 14 (± 1)	45 (72.6)	38 (65.5)	0.402
Day 21 (± 1)	52(83.9)	39 (67.2)	0.033*
***Vomiting***			
Baseline	47 (75.8)	51 (87.9)	0.086
Day 1	37 (59.7)	42 (72.4)	0.142
Day 2	39 (62.9)	32 (55.2)	0.389
Day 3	35 (56.5)	32 (55.2)	0.888
Day 7 (± 1)	48 (77.4)	40 (69.0)	0.295
Day 10 (± 1)	51 (82.3)	42 (72.4)	0.197
Day 14 (± 1)	51 (82.3)	45 (77.6)	0.523
Day 21 (± 1)	57 (91.9)	48 (82.8)	0.214

***c. Variation of CINV degree evaluated by CTCAE in each group***			
	Mean (SD) True acupuncture group (n = 62)	Sham acupuncture group (n = 58)	*P* value
***Nausea***			
Day 1	0.258 (0.599)	0.259 (0.609)	0.996
Day 2	0.355 (0.546)	0.517 (0.755)	0.182
Day 3	0.306 (0.616)	0.621 (0.834)	0.021*
Day 7 (± 1)	0.032 (0.724)	0.310 (0.654)	0.030*
Day 10 (± 1)	− 0.194 (0.649)	0.086 (0.539)	0.011*
Day 14 (± 1)	− 0.258 (0.676)	0.000 (0.375)	0.011*
Day 21 (± 1)	− 0.387 (0.662)	− 0.017 (0.477)	0.001*
***Vomiting***			
Day 1	0.226 (0.556)	0.138 (0.348)	0.298
Day 2	0.210 (0.631)	0.448 (0.776)	0.068
Day 3	0.210 (0.604)	0.414 (0.676)	0.085
Day 7 (± 1)	0.000 (0.444)	0.190 (0.512)	0.033*
Day 10 (± 1)	− 0.097 (0.503)	0.207 (0.585)	0.003*
Day 14 (± 1)	− 0.081 (0.552)	0.155 (0.556)	0.021*
Day 21 (± 1)	− 0.194 (0.507)	0.069 (0.454)	0.003*

*CINV* Chemotherapy Induced Nausea and Vomiting, *CTCAE* the Common Terminology Criteria for Adverse Events, *SD* standard deviation

*P values are based on independent-samples t tests comparing differences in between-group means

**Fig. 2:**
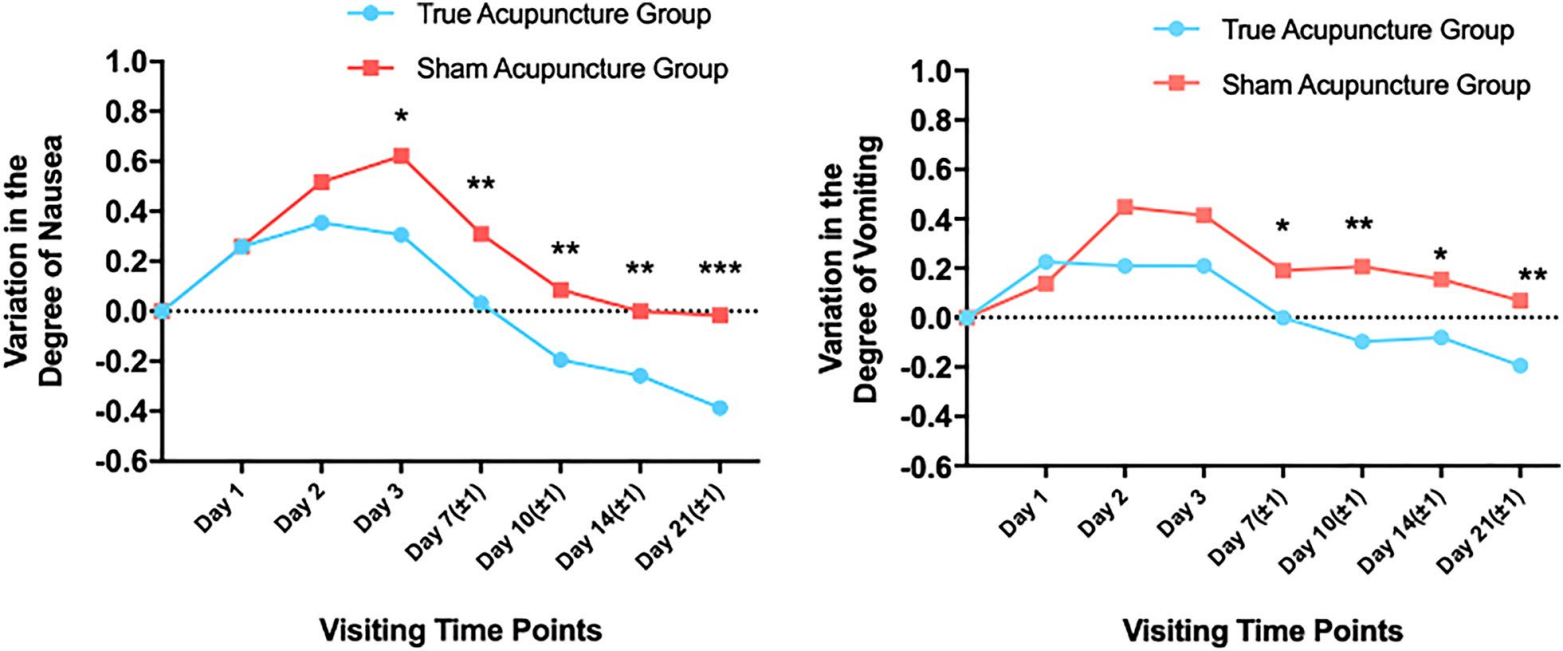
Observed and Fitted Group Variation of CINV Degree Evaluated by CTCAE in Both Groups

We evaluated the variation of nausea and vomiting degrees with CTCAE4.0 in both groups (Table 2c). Original nausea and vomiting degrees between groups showed no significant statistical difference. The nausea degree in the true acupuncture group increased in the first 2 days and reached a peak on day 2 by 0.355 points compared to baseline, and then decreased gradually till the end of the chemotherapy circle. There was a similar tendency in the nausea variation of the sham acupuncture group except that the value keeps increasing to day 3 by 0.621 points. The difference between the two groups had statistical significance since day 3 (0.306 vs. 0.621, *P *= 0.021) until the end of observation (Fig. 2). The rise in the true acupuncture group at the beginning of chemotherapy was lower than that in another group, which indicating true acupuncture relieve the severity of nausea during the delay CINV and follow-up periods.

Likewise, the variation trend of vomiting in the sham acupuncture group was similar to that in nausea assessment. The vomiting degree rose by 0.448 points on day 2 and then went down gradually. The degree variation in the true acupuncture group continually decreased in the observation. And the values in the true acupuncture group were lower than those in the control group. The differences between the two groups were significant since day 7(0 vs. 0.19, *P *= 0.033). It demonstrated that there was no significant difference during the CINV period, but true acupuncture could relieve symptoms in the follow-up period.

## Secondary outcomes

During the observation, the nutritional conditions of patients were evaluated with the SNAQ scale (Table 3). The score of SNAQ decreased on day 7 and slightly rose after that. The true acupuncture group had a higher score compared to the sham acupuncture group in the follow-up duration, and the difference was significant on day 14 (21.82 vs.20.12, *P *= 0.003) and day 21 (22.39 vs. 20.43, *P *= 0.001). It depicted that patients receiving true acupuncture would have better nutritional status after chemotherapy.

**Table 3:** Observed and Fitted Group Results for Simplified Nutritional Appetite Questionnaire assessment

	True acupuncture group (n = 62)		Sham Acupuncture Group (n = 58)		*P*-value
	Mean (SD)	95% CI	Mean (SD)	95% CI	
Day 1	20.145 (3.463)	19.27 ~ 21.02	21.138 (2.453)	20.49 ~ 21.78	0.071
Day 7 (± 1)	19.113 (3.052)	18.34 ~ 19.89	18.983 (3.301)	18.11 ~ 19.85	0.823
Day 10 (± 1)	20.806 (3.274)	19.98 ~ 21.64	19.897 (2.942)	19.12 ~ 20.67	0.113
Day 14 (± 1)	21.823 (3.262)	20.99 ~ 22.65	20.121 (2.728)	19.40 ~ 20.84	0.003*
Day 21 (± 1)	22.387 (3.138)	21.59 ~ 23.18	20.431 (2.872)	19.68 ~ 21.19	0.001*

*SD* standard deviation, *CI* confidence interval

*P values are based on independent-samples t-tests comparing differences in between-group means

While assessing the anxiety and depression of patients, no significant difference was found between two groups (Data were shown in Additional file 2: Table S2). All participants’ ECOG score ranged from 0 to 2.

## Safety assessments

Safety was evaluated in 125 participants who were randomly enrolled and received at least 3 days of study intervention (Data were shown in Additional file 2: Table S3). Two participants in the true acupuncture group and one in the sham acupuncture group were not evaluable who withdrew consents without any intervention. One participant in the true acupuncture group and 5 in the sham acupuncture group discontinued the treatment due to transferring to other hospitals for a therapeutic reason. During the observation, 4 patients reported headache in the true acupuncture group. Diarrhea (n = 2), constipation (n = 2), and headache (n = 4) were reported in the sham acupuncture group. One participant was evaluated with increased Urea nitrogen and Creatinine related to primary renal disease, while one in the sham acupuncture group with slightly ALT increased. No apparent adverse events related to acupuncture were observed. Patients who need an additional rescue for CINV were 17 (26.1%) and 20 (33.3%) in the true or sham acupuncture group, respectively (*P *= 0.435, data were not shown in the table).

## Discussion

We conducted a multi-center, single-blind, sham-controlled prospective clinical research to investigate the effect of acupuncture in preventing chemotherapy-induced nausea and vomiting. There is no noticeable improvement of true acupuncture in the complete response rate of CINV compared to the sham-control. However, TA indeed remarkedly alleviated the severities of nausea (day-3 to day-21) and emesis (day-7 to day-21) induced by chemotherapy. TA also contributed to nutritional status recovery since the day-14 after chemotherapy initiation.

Nowadays, CINV is less of a challenge for patients receiving chemotherapy owing to the development of antiemetic medications. We still need to pay special attention to the CINV occurred unreported by the data. The limitations of traditional antiemetic trials conclude evaluating outcomes only after the first circle of chemotherapy and mainly focusing on the cisplatin-based chemotherapy regimen. These designs are critical for the preciseness of researches, but also confine the application of the conclusions. Patients undergoing other drugs or multi-day chemotherapy regimens, or receiving multicycle treatments remain faced with a higher risk of CINV. Also, CINV is affected by several risk factors, such as a history of nausea/vomiting, female sex, the expectancy of CINV, younger age, anxiety, history of morning sickness, and low alcohol intake [22]. Antiemetic prophylaxis based on a risk model guide led to improved control effect of acute and delayed CINV [23]. Nevertheless, these factors are not much considered in clinical application. Compared to emesis, nausea is a more distressing symptom and less controlled [8]. More emphasis should be put on alleviating nausea to improve the QOL of cancer patients. It will promote the psychological and physical wellness of patients. Adjunctive measures require better management of CINV.

In our study, we didn’t find superior CRR in the TA group but observed that TA reduced the severities of CINV and augmented the score of nutritional status. The nausea degree increased by 0.306 in the TA group while 0.621 in the SA group (*P *= 0.021) on day 3. Although there was no significant improvement in vomiting in the 120 h after chemotherapy initiation, TA reduced the vomiting degree since day 7(0 vs.0.190, *P *= 0.033). These results are consistent with findings by other researchers [24, 25]. Acupuncture didn’t exhibit a promising effect in reducing the occurrence of CINV, which might due to acupuncture have less influence on preventing CINV from taking place than antiemetic drugs. The impact of acupuncture took a longer time to show up and mainly manifested in modulating gastrointestinal functions. The mechanisms related to electroacupuncture were analyzed with animal models. Electroacupuncture at Neiguan (PC 6) and Jianshi (PC 5) could reduce nausea and vomiting during chemotherapy, which was possibly associated with the deduction of 5-HT_3_ and dopamine [26]. It was also confirmed that electro-acupuncture(EA) at Zhongwan (CV12) alleviated the cisplatin-induced anorexia in rats by reducing the levels of plasma monoamine neurotransmitters 5-hydroxytryptamine, 5-hydroxyindoleacetic acid, dopamine, and norepinephrine; as well as upregulating the expression of ghrelin and neuropeptide Y [27]. In another rat model, EA at CV12 not only attenuated the cisplatin-induced increase in 5-HT but also suppressed neuronal activation marker c-Fos expression in the nucleus tractus solitarius (NTS) [28]. Acupuncture exhibits a positive effect on alleviating nausea and promoting the nutrition intake, which compensates for the deficiency of antiemetic medication. Our results also indicated that acupuncture functioned after a period of time of treatment in general cases. It might be an effective strategy to initiate acupuncture ahead of chemotherapy for patients with a high risk of CINV. What’s more, for the sake of widespread implementation, our research allocated patients with multiple types of advanced cancers receiving various regimens, multi-circle of chemotherapy. TA and SA groups were comparable with similar participant composition with each factor. However, it is not sufficient to draw a solid conclusion for which population is more suitable to receive acupuncture during chemotherapy. An enriched enrollment strategy was indicated for future studies.

To eliminate the impact of the subjective factor, patients in both groups received acupuncture treatment with the same frequency, needle-retained time, an interval of treatment to achieve effective masking. Investigators in charge of participant interviews were also blind to the assignment. Whereas, the principal investigator who was responsible for distributing tasks and acupuncturists were not blinded. We also applied blind evaluation in statistical analysis. All these measures contributed to addressing reliable conclusions.

## Strengths and Limitations

This study has several strengths. The intervention was conducted by several acupuncturists undergoing rigorous standardized training to make sure the operational consistency in this trial. Patients were treated at multiple sites with different stages of cancer, different cycles of chemotherapy, which more like the situations in the real world and increased the generalizability of the findings. The observation not only for the CINV phase (5 days since chemotherapy administration) but also including the follow-up 16 days. It made it possible to evaluate the effect of acupuncture in the whole chemotherapy cycle.

This study also has several limitations. First, in the study design, waitlist control was in need to eliminate the influence of the physiological effect of sham acupuncture [29]. Second, some participants in the study withdrawing for different reasons might impact the evaluation of the effects of acupuncture. Third, enrollment progressed slower than expected due to the patients’ turnover in the hospitals, limiting the enthusiasm of patients for participating in a randomized clinical trial. Fourth, there may exist the possibility that rare patients were aware of their allocation due to possessing a good knowledge of Chinese Medicine.

Article Summary—Strengths and limitations of this study

1. Acupuncturists and researchers received rigorous standardized training which guaranteed the operational consistency and the results reliability of the study.
2. The participants met the inclusion criteria of diversity increased the generalizability of the findings.
3. The waitlist control was in need to eliminate the influence of the physiological effect of sham acupuncture.

## Conclusion

In conclusion, the current study found the effect of acupuncture in preventing CINV occurring is relatively modest. Still, it was feasible and practical to apply acupuncture for alleviating chemotherapy-induced nausea and vomiting and improving nutritional status of patients with advanced cancer.

## Supplementary information

**Additional file 1.** Study protocol.

**Additional file 2.** Acupuncture therapy regimens and supplementary results.

## Data Availability

All relevant data are within the paper and its supporting information files.

## Abbreviations

CINV: Chemotherapy-Induced Nausea and Vomiting
CTCAE: The common terminology criteria for adverse events
ECOG: The eastern cooperative oncology group score
SNAQ: Simplified nutritional appetite questionnaire
HADS: Hospital anxiety and depression scale
HEC: Highly emetogenic chemotherapy
MEC: Moderately emetogenic chemotherapy
NCI: National cancer institute
ZPS: Zubrod score
AEs: Adverse events
SPSS: Statistical package of social sciences software
QOL: Quality of life
5-HT_3_: 5-Hydroxytryptamine type 3
RAs: Receptor antagonists
NTS: The nucleus tractus solitarius
NK_1_: Neurokinin-1
