# Supplementary material for: Efficacy of acupuncture in the prevention and treatment of chemotherapy-induced nausea and vomiting in patients with advanced cancer: a multi-center, single-blind, randomized, sham-controlled clinical research
Source: Chin Med. 2020 Jun 3;15:57. doi: 10.1186/s13020-020-00333-x (PMC7268447; doi:10.1186/s13020-020-00333-x)
Supplement: Supplementary file 1 — Additional file 1. Study protocol. [file 13020_2020_333_MOESM1_ESM.pdf]

Li et al. Trials (2017) 18:185
DOI 10.1186/s13063-017-1927-2

Trials

STUDY PROTOCOL Open Access

Effect of acupuncture in prevention and treatment of chemotherapy-induced nausea and vomiting in patients with advanced cancer: study protocol for a randomized controlled trial

Qi-wei Li[1,2], Ming-wei Yu[1], Guo-wang Yang[1], Xiao-min Wang[1*], Huan Wang[1], Chen-xi Zhang[1], Na Xue[1], Wei-ru Xu[1], Qi Fu[1], Zhong Yang[1] and Lin Yang[1]

Abstract

Background: Chemotherapy-induced nausea and vomiting (CINV) is one of the most common and distressing side effects in patients with cancer. The introduction and development of antiemetic drugs have significantly improved the ability of clinicians to control CINV, but it is not easy to translate to practical application, owing to financial issues, provider-related barriers, and patient factors. Nondrug therapies are needed to alleviate the symptoms of CINV. Acupuncture is an appropriate adjunctive treatment for CINV, but additional evidence is needed.

Methods/design: This study is a multicenter, randomized, sham-controlled prospective clinical trial. A total of 136 participants will be randomly allocated into the intervention group (verum acupuncture) or the control group (sham acupuncture) in a 1:1 ratio. All treatment will be given for 5 days. Participants in both groups will receive acupuncture sessions twice on the first day of chemotherapy and once consecutively on the following 4 days. Each session takes approximately 30 minutes. The primary outcome measure will be the Common Terminology Criteria for Adverse Events to assess CINV. The secondary outcome measures will be the Eastern Cooperative Oncology Group score, Simplified Nutritional Appetite Questionnaire, and Hospital Anxiety and Depression scale. Safety will be assessed at each visit.

Discussion: The results of this trial will provide clinical evidence for the effect and safety of acupuncture for CINV.

Trial registrations: ISRCTN Registry identifier: ISRCTN13287728). Registered on 28 February 2015. ClinicalTrials.gov identifier: NCT02369107. Registered on 17 February 2015.

Keywords: Chemotherapy-induced nausea and vomiting, Acupuncture, ECOG, SNAQ, CTCAE, Sham acupuncture, Clinical research trial

* Correspondence: wangxiaomin_bhtcm@126.com
[1]Beijing Hospital of Traditional Chinese Medicine affiliated with Capital Medical University, No. 23, Back Road of Art Gallery, Dong Cheng District, Beijing 100010, China
Full list of author information is available at the end of the article

© The Author(s). 2017 **Open Access** This article is distributed under the terms of the Creative Commons Attribution 4.0 International License (http://creativecommons.org/licenses/by/4.0/), which permits unrestricted use, distribution, and reproduction in any medium, provided you give appropriate credit to the original author(s) and the source, provide a link to the Creative Commons license, and indicate if changes were made. The Creative Commons Public Domain Dedication waiver (http://creativecommons.org/publicdomain/zero/1.0/) applies to the data made available in this article, unless otherwise stated.

Background

Chemotherapy-induced nausea and vomiting (CINV) is one of the most common and distressing side effects in patients with cancer. Without prophylactic medications, more than 90% patients undergoing highly emetogenic chemotherapy (HEC) and approximately 30% to 90% of patients undergoing moderately emetogenic chemotherapy (MEC) will experience CINV [1]. The most important influencing factor for CINV is the chemotherapy regimen. The severity and incidence of CINV are usually determined by chemotherapeutic agent, dosage, combinations with other treatment approaches, and patient characteristics [2–4]. Patient-related variables that influence the risk of CINV include age (persons younger than 50 years old tend to experience more CINV), sex (females have a high risk of CINV), history of low prior chronic alcohol intake, history of previous chemotherapy-induced emesis, and others [5].

CINV decreases patients' quality of life (QOL) and impacts their physical activities and social functioning. Uncontrolled CINV may result in dehydration as well. These disadvantages may lead to deferring or canceling treatment. Thus, patients will miss the best time for treatment and face limited therapeutic effect. Uncontrolled CINV may increase unnecessary healthcare costs, aggravate burden on medical and nursing resources, and prolong hospitalizations [6, 7].

The introduction and development of antiemetic drugs have significantly improved the ability of clinicians to control CINV. The mainstays of antiemetic therapy include serotonin (5-HT_3) receptor antagonists (RAs) and neurokinin 1 (NK-1) RAs [1]. These agents block receptors for serotonin and substance P, which are two key neurotransmitters that participate in the physiopathological changes of vomiting [8]. Palonosetron, a second-generation 5-HT_3 RA, has demonstrated benefits in controlling both acute and delayed CINV (78.1% and 65.6%, respectively) associated with HEC or MEC [9]. The agents including NK-1 RA and palonosetron perform better in the delayed phase than do other 5-HT_3 RAs, which suggests that different mechanisms may be involved between acute and delayed CINV. Although the availability of effective management strategies has been recognized, it is not easy to translate them to practical application, owing to financial issues, provider-related barriers, and patient-related factors (e.g., adherence and underreporting of CINV) [10, 11]. Caution should be taken when the drug is used during chemotherapy treatment because of the risks for headache, fatigue, and constipation, which are liable to add to the suffering of patients with advanced cancer.

Researchers and patients are seeking additional methods of controlling CINV, such as nondrug therapies. Acupuncture, as a typical treatment modality of traditional Chinese medicine (TCM), has been applied for diseases for over 2000 years. A growing interest in TCM can be noted in that researchers from many countries have conducted clinical trials to evaluate the safety and effectiveness of acupuncture in managing CINV. Several studies have drawn encouraging conclusions on the efficacy of acupuncture in CINV [12, 13]. Rithirangs-riroj et al. [12] found that acupuncture was effective in preventing delayed CINV and promoting QOL. With fewer adverse effects, it may be used as an alternative treatment option for CINV. Shen et al. [13] conducted a three-arm, parallel-group, randomized controlled study and showed that in patients with breast cancer receiving high-dose chemotherapy, adjunct electroacupuncture was more effective in controlling emesis than minimal needling or antiemetic pharmacotherapy alone, although the observed effect had limited duration. Therefore, acupuncture could be considered as a supplementary treatment for patients experiencing CINV.

On the basis of a systematic review [14], acupuncture could be an appropriate adjunctive treatment for CINV; however, additional studies are needed because among 11 studies of the efficacy of acupuncture for CINV, only 1 study had a low risk of bias (ROB). Therefore, we plan to perform a multicenter randomized controlled trial to investigate the efficacy and safety of acupuncture in the prevention and treatment of CINV in patients with advanced cancer.

Methods/design

Study design

This study is a multicenter, randomized, sham-controlled prospective clinical trial aimed at estimating the efficacy and safety of acupuncture on CINV by comparing a verum acupuncture group with a sham acupuncture group. The participants who are considered suitable for the study will be randomly allocated into the intervention group (verum acupuncture) or the control group (sham acupuncture) in a 1:1 ratio.

Study participants

Population

A target sample of 136 participants will be recruited from three hospitals (Beijing Shijitan Hospital, Beijing Friendship Hospital, and Beijing Hospital of Traditional Chinese Medicine) by using posters and website advertisements. Informed consent will be obtained from all participants before randomization.

Sample size

According to the previous pilot study, the complete control rates of nausea and vomiting are 32% and 59% [15], respectively. In the intervention group, the complete control rate is anticipated to rise by 25%. On the basis of

0.8 power to detect a significant difference ($\alpha = 0.05$, two-sided) and allowing for a 20% withdrawal rate, we plan to enroll a total of 136 participants, with 68 participants in each group.

Recruitment of participants

Several strategies will be applied to participant recruitment. In this trial, we will recruit mainly participants who are hospitalized patients at the three hospitals mentioned above. Posters will be put up in the hospitals to inform patients and their family members of the study, and advertisements will be placed across WeChat and hospital websites. The posters and advertisements contain brief introductions about the population needed to be enrolled, the free acupuncture treatments offered to participants, and contact information of the researchers.

Baseline assessment

A baseline registration will be undertaken before treatment. At baseline assessment, participants will provide a review of malignancy history, including operation history, pathology, and history of prior treatment. A safety evaluation will be performed as well.

Inclusion criteria

Patients who meet all the following requirements will be allowed to enroll:

1. Patients with definite pathological diagnosis of lung cancer, breast cancer, or gynecological cancer
2. Aged 18–75 years
3. Patients who will receive chemotherapy treatment, the regimen of which contains cisplatin, anthracycline, or taxane during the study period
4. Eastern Cooperative Oncology Group (ECOG) score between 0 and 2
5. Expected lifetime longer than 6 months
6. Willing to participate in the study and sign the consent forms

Exclusion criteria

Patients who meet all the following criteria will be excluded:

1. Patients with severe cardiac arrhythmia, serious hepatorenal abnormal function (glutamic oxaloacetic transaminase, glutamic-pyruvic transaminase, or total bilirubin three times higher than normal or blood urea nitrogen or urine creatinine two times higher than normal), or immune system or hematopoietic system diseases
2. Pregnant or lactating women
3. Patients with intractable vomiting caused by malignant brain metastases, intracranial hypertension, digestive tract obstruction, severe liver or renal dysfunction, brain tumors, cerebrovascular diseases, or other reasons
4. Patients with coagulopathy, thrombocytopenia, or other bleeding disorders
5. Patients definitely diagnosed with depression, anxiety disorder, and/or psychosis
6. Patients with sepsis or bacteremia
7. Patients with lymphedema in acupuncture stimulation area
8. Patients who are afraid of acupuncture stimulation or are allergic to stainless steel needles

Withdrawal from the study

Participants will be allowed or asked to withdraw from the study in the following circumstances:

1. They would prefer not to be subjected to the assigned treatment for various reasons at any stage of the trial.
2. Adverse events (AEs) occur that necessitate their withdrawal from the trial.
3. They cannot fully participate in the treatment or follow-up period.
4. Patients can withdraw from the study voluntarily for any reason.

Randomization and blinding

A total of 136 participants will be randomly allocated into the intervention group or the control group. Beijing Shijitan Hospital and Beijing Friendship Hospital will each recruit 36 patients. Beijing Hospital of Traditional Chinese Medicine will recruit 64 patients. In this study, we will carry out block randomization in a 1:1 ratio according to the sequence generated with SAS version 9.1.3 software (SAS Institute Inc., Cary, NC, USA). Randomly allocated information will be sealed in opaque envelopes and delivered to each center. Random allocation will be performed after eligible participants consent in written form and complete baseline assessments. The investigators and practitioners will be aware of the allocated group, but the outcome assessors and data analysts will not (Fig. 1).

The research assistant responsible for randomization and acupuncturists is not blinded to the participant allocation. It is not possible to blind participants, owing to the different acupuncture points of the two groups. Blinding will be applied to the outcome assessment.

Procedure

According to the predetermined randomization envelopes, participants will be randomly allocated to the verum or sham acupuncture group. Participants will be interviewed in person or on the phone. During the study

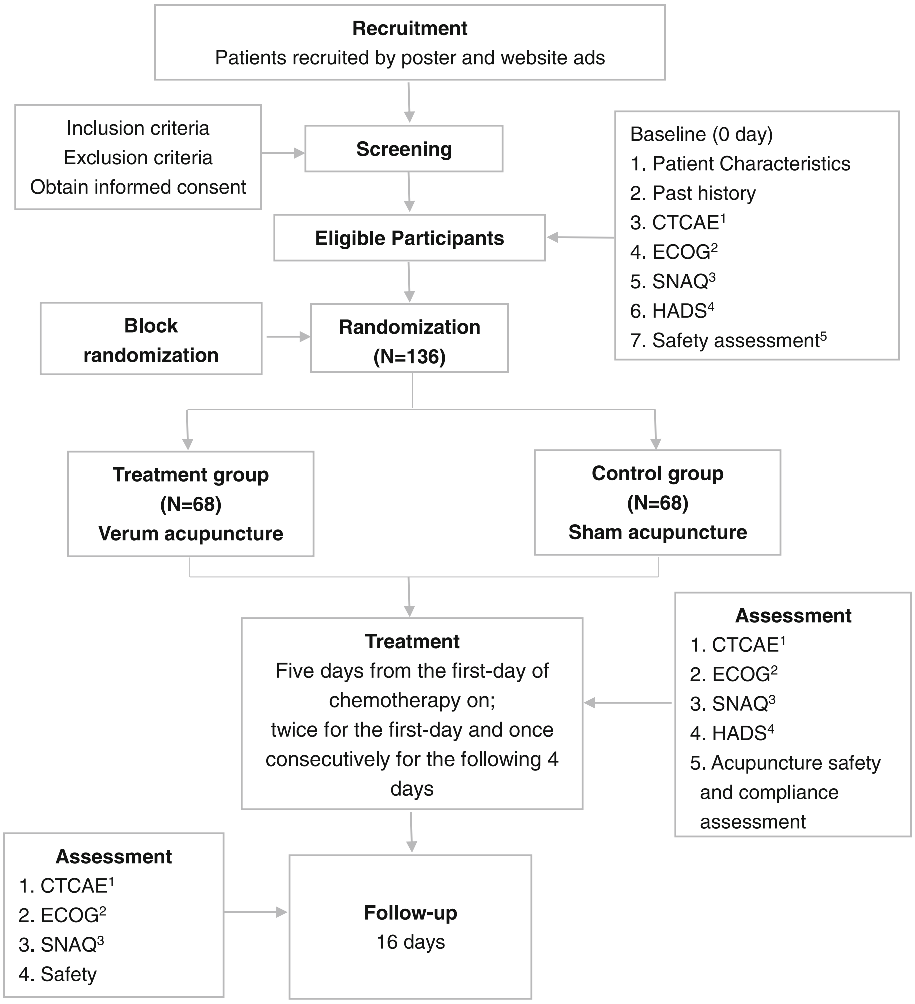

Fig. 1 Project overview. The safety assessment comprises a routine blood test, routine urine test, routine feces test, kidney function test, liver function test, and electrocardiogram. *CTCAE* Common Terminology Criteria for Adverse Events (Chinese version), *ECOG* Eastern Cooperative Oncology Group score (Chinese version), *SNAQ* Simplified Nutritional Appetite Questionnaire, *HADS* Hospital Anxiety and Depression Scale (Chinese version)

period, participants will be followed by blinded telephone interviewers and be asked some questions on their CINV symptoms, appetite, and nutritional status, which will be recorded on the case report forms.

Interventions

All participants will go through a standardized interview and receive more information about this study. To minimize treatment bias, all the acupuncturists who participate in this trial are specialists in acupuncture. They have more than 3 years of experience in acupuncture treatment and have obtained acupuncture licenses (Chinese medicine practitioner license), which are provided by the Ministry of Health of the People's Republic of China. Before performing this trial, all acupuncturists will receive special training regarding the purpose and content of the trial, treatment strategies, and quality control. We have followed the Standard Protocol Items: Recommendations for Interventional Trials (SPIRIT) 2013 statement, which defines standard protocol items for clinical trials [16] (*see* Additional file 1). The treatments are fully documented according to good clinical practice guidelines.

The trial will last for 21 days, including treatment and follow-up periods. All participants will receive treatment for 5 days (from the first day of chemotherapy). The schedule of enrollment, interventions, and assessments is provided in Fig. 2. Participants in both groups will receive verum or sham acupuncture sessions twice on the first day of chemotherapy and once each on the next consecutive 4 days. Each acupuncture session takes approximately 30 minutes. The number of the needles used will be ten in each session for both groups. Sterile needles for single use (Ande, Guizhou, China) will be

STUDY PERIOD										
	Enrolment Allocation	Post-allocation								Close-out
TIMEPOINT (day)	-7~0	1	2	3	4	5	7 (±1)	10 (±1)	14 (±1)	21 (±1)
Eligibility screen	X									
Informed consent	X									
Allocation	X									
Demographics	X									
Medical history	X									
INTERVENTIONS:										
Verum acupuncture		◆———	———	———	———	———◆				
Sham acupuncture		◆———	———	———	———	———◆				
ASSESSMENTS:										
CTCAE[1]	X	X	X	X			X	X	X	X
ECOG[2]	X			X			X		X	X
SNAQ[3]	X			X			X		X	X
HADS[4]	X			X			X			
SAFETY ASSESSMENT[5]	X	X	X	X					X	X

Fig. 2 The schedule of enrollment, interventions, and assessments. The safety assessment comprises a routine blood test, routine urine test, routine feces test, kidney function test, liver function test, and electrocardiogram. *CTCAE* Common Terminology Criteria for Adverse Events (Chinese version), *ECOG* Eastern Cooperative Oncology Group score (Chinese version), *SNAQ* Simplified Nutritional Appetite Questionnaire, *HADS* Hospital Anxiety and Depression Scale (Chinese version)

used in this study. Twenty-five-gauge (0.25 mm in diameter), 40-mm-long needles are used for both groups at the limbs and abdomen. Needles in the intervention group will be inserted 10–35 mm in depth and manually manipulated by rotation methods to produce a characteristic sensation known as De Qi, in which a feeling of needle sensation refers to tenseness around the needle felt by the acupuncturist and numbness, distention, soreness, and heaviness around the point felt by the patient), in addition to electroacupuncture. During the verum acupuncture session, acupuncturists will add manipulation to the needles every other 10 minutes. In the control group, needles will be inserted about 1–2 mm in depth with no manipulation. Participants in both groups will receive intravenous ondansetron (ondansetron hydrochloride injection; Ningbo Team pharm Co., Ltd., Ningbo, China) 8 mg twice daily as a foundation antiemetic regimen.

Acupoints used in the intervention group

The stimulation points in the intervention group are RN12 (Zhongwan), LR13 (Zhangmen, bilaterally), RN6 (Qihai), ST25 (Tianshu, bilaterally), PC6 (Neiguan, bilaterally), and ST36 (Zusanli, bilaterally). All these acupuncture points except ST36 will be stimulated manually every 10 minutes. A battery-operated SDZ-II Electronic Acupuncture Treatment Instrument (Hua Tuo Medical Instruments Co., Ltd., Suzhou Jiangsu, China) will be connected to the needle inserted in ST36 and a skin electrode located at ST36 laterally about 5 cm on both sides. Electrical stimulation will be delivered for 30 minutes at alternating frequencies of 2 and 100 Hz, within an intensity of each patient's maximum tolerance.

Sham acupoints used in the control group

To make the quantity of stimulus uniform between two groups, the decision was made to use the same kind, size, and number of needles for the control group as for the intervention group. The stimulation points do not belong to TCM meridians. The acupoints selected in both groups are listed in Table 1.

Needles will be inserted to a depth of about 1–2 mm and retained in place for 30 minutes. No manual manipulation will be given. The sham acupuncture points are chosen from three different areas on the body (the abdomen, the inside of the upper limb, and the lateral

Table 1 Acupuncture points and needle stimulation methods in treatment group

Acupuncture point	Stimulation method	Depth of insertion
RN12 (Zhongwan)	Reducing twirling	Perpendicular needling 2.5–3.5 cm
LR13(Zhangmen, bilaterally)	Reducing twirling	Oblique or perpendicular needling 1.3–2.5 cm
RN6 (Qihai)	Tonic twirling	Perpendicular needling 2.5–3.5 cm
ST25 (Tianshu, bilaterally)	Reducing twirling	Perpendicular needling 2.5–3.5 cm
PC6 (Neiguan, bilaterally)	Reducing twirling	Perpendicular needling 1.3–2.5 cm
ST36 (Zusanli, bilaterally)	Tonic twirling with electrical stimulation	Perpendicular needling 2.5–3.5 cm

lower leg), which do not correspond to recognized acupuncture points and are deemed to have no therapeutic value.

Outcome measures

Primary outcome measures

CTCAE 4.0 The Common Terminology Criteria for Adverse Events (CTCAE 4.0), formerly called the *Common Toxicity Criteria*, is a set of criteria for the standardized classification of adverse effects of drugs used in cancer therapy. The CTCAE system is established by the National Cancer Institute. It includes the standard to assess nausea and vomiting. It will be used at baseline, day 1, day 2, day 3, day 7 (±1), day 10 (±1), day 14 (±1), and day 21 (±1). Patients have daily questionnaires to fill in that will be used to record the frequency and extent of CINV during the observation period.

Secondary outcome measures

ECOG The ECOG score, also called the *World Health Organization score* or *Zubrod Performance Status score*, runs from 0 to 5, with 0 denoting perfect health and 5 denoting death [17]. The ECOG score is used to assess performance status of patients.

HADS The Hospital Anxiety and Depression Scale (HADS) was originally developed by Zigmond and Snaith [18]. The HADS is a 14-item scale that generates ordinal data in which 7 of the items relate to anxiety and 7 relate to depression. It is commonly used to estimate the degree of anxiety and depression that a patient is experiencing.

SNAQ The Simplified Nutritional Appetite Questionnaire is a self-assessment nutritional screening tool that predicts weight loss and could be used to screen patients at risk of malnutrition or malnourishment [19].

These measures (except HADS) will be assessed at baseline, day 3, day 7 (±1), day 14 (±1), and day 21 (±1). The HADS will be evaluated at baseline, day 3, and day 7 (±1). Patients will be asked to finish questionnaires designed by the researcher. The detailed outcome assessment time points are provided in Table 2.

Safety assessment

To exclude any related serious diseases, before randomization, patients will be asked to undergo routine tests of blood, urine, and stool; an electrocardiogram; a liver function test; and a kidney function test. These tests will be performed at the 14th day and the 21st day of the study to evaluate the safety of this trial. During the chemotherapy period, patients may be more prone to develop adverse reactions such as thrombocytopenia,

Table 2 Data collection schedule

	Screening	Treatment			Follow-up			
Visit number	1	2	3	4	5	6	7	8
Day number	−14 to 0	1	2	3	7 ± 1	10 ± 1	14 ± 1	21 ± 1
Patient characteristics	X							
Past history	X							
ECOG	X		X		X		X	
CTCAE	X	X	X	X	X	X	X	X
SNAQ	X			X	X		X	X
HADS	X			X	X			
Safety assessment[a]	X						X	X
Combined medication	X	X	X	X	X	X	X	X
Adverse events		X	X	X	X	X	X	X
Acupuncture safety assessment		X	X	X	X			
Compliance		X	X	X	X			

Abbreviations: CTCAE Common Terminology Criteria for Adverse Events (Chinese version); *ECOG* Eastern Cooperative Oncology Group (Chinese version); *SNAQ* Simplified Nutritional Appetite Questionnaire; *HADS* Hospital Anxiety and Depression Scale (Chinese version)
[a] Safety assessment includes a routine blood test, routine urine test, routine feces test, kidney function test, and liver function test

myelosuppression, and hemoglobin decrease. If any AEs occur during the trial, patients will be treated as soon as possible. Researchers will record drug combinations, including the drug's name, frequency of use, dosage form, and the dose. Additional antiemetic agents will be included in the record of drug combinations.

Statistical analysis

Statistical analysis will be performed with IBM SPSS Statistics version 19.0 software (IBM, Armonk, NY, USA). First, the baseline characteristics of the two groups, such as age, sex, and chemotherapy regimen, will be analyzed by an unpaired t test or paired t tests. The second step is to compare the efficacy of acupuncture in the prevention and treatment of CINV in both groups. The average intensity of nausea and vomiting and the HADS score will be tested for normal distribution. If the data follow a normal distribution, a two-samples (independent) t test will be used. Otherwise, the data will undergo Wilcoxon testing. A P value less than 0.05 will be considered statistically significant.

Efficacy and safety analyses will be conducted in accordance with the intention-to-treat principle. Missing values will be imputed by the last observation carried forward method.

Discussion

Acupuncture, one of the major components of TCM, is deemed to be an available complementary therapy to relieve symptoms. The efficacy of acupuncture has been proved by many randomized controlled trials [12, 13]. Authors of a systematic review demonstrated that acupuncture was an appropriate adjunctive therapy for CINV, but the supporting evidence was not sufficient, because most of the randomized controlled trials analyzed had high or unclear ROB [14]. These methodological and practical limitations included small sample size, greater-than-anticipated withdrawal, lack of a control group, and unclear statements about randomized allocation. Therefore, more powerful evidence is still needed to verify the efficacy and safety of acupuncture for CINV. We have presented the design of a randomized controlled trial of verum acupuncture compared with sham acupuncture. Completion of this trial will contribute to verifying the efficacy of acupuncture for the treatment of CINV.

However, this trial has several methodological and practical limitations. Previous randomized controlled trials showed that there is no significant difference in statistical analysis between verum and sham acupuncture groups. Shallow insertion may also lead to latent physiological effects to alleviate the symptoms. In this trial, the sham points are selected to avoid meridians and acupoints, but we cannot rule out physiological effects from the control group design. Another limitation is that the research assistant and therapists are not blinded in this study. It is almost impossible to have genuine double blinding in acupuncture trials, because acupuncturists are responsible for controlling participants' perception of needling. We cannot distinguish effectiveness from placebo effects and physiological effects. A bias due to unblinding cannot be excluded.

Trial status

This trial is currently recruiting participants up to april 2017, the study has recruited 118 patients. The first participant was enrolled on 13 March 2015.

Additional file

Additional file 1: SPIRIT 2013 checklist: recommended items to address in a clinical trial protocol and related documents. (DOCX 52 kb)

Abbreviations

AE: Adverse event; CINV: Chemotherapy-induced nausea and vomiting; CTCAE: Common Terminology Criteria for Adverse Events; ECG: Electrocardiogram; ECOG: Eastern Cooperative Oncology Group; HADS: Hospital Anxiety and Depression Scale; HEC: Highly emetogenic chemotherapy; 5-HT_3: Serotonin; MEC: Moderately emetogenic chemotherapy; NK-1: Neurokinin 1; QOL: Quality of life; RA: Receptor antagonist; ROB: Risk of bias; SNAQ: Simplified Nutritional Appetite Questionnaire; SPIRIT: Standard Protocol Items: Recommendations for Interventional Trials; TCM: Traditional Chinese medicine

Acknowledgements

We thank Nan Peng, Chen Li, and Yi Zhang from the Capital Medical University for kind assistance with this project. We thank Nan Hu, Ming-wen Zhao and Yan-da Xie from Beijing University of Chinese Medicine for performing the acupuncture operation. We also extend thanks to Yong-ming Zhou and Dao-Rui Li for recruiting patients. Finally, we thank all the other people who have been involved in the study.

Funding

This work is financed by grants from Beijing Municipal Administration of Hospitals (number XM201410).

Availability of data and materials

Not applicable.

Authors' contributions

MY and XW conceived of and designed the trial. QL drafted the manuscript. GY provided critical contributions. NX, WX, LY, QF, and ZY will enroll the participants. QL, HW, and CZ will participate in recruiting patients and collecting the data. All authors read and approved the final manuscript.

Competing interests

The authors declare that they have no competing interests.

Consent for publication

Not applicable.

Ethics approval and consent to participate

The protocol is being carried out according to good clinical practice guidelines and in accordance with the principles of the Declaration of Helsinki. This study was approved by the research ethics committee of Beijing Hospital of Traditional Chinese Medicine Affiliated to Capital Medical University on 19 November 2014 (reference 2014BL-067). All participants will give written informed consent.

Publisher's Note

Springer Nature remains neutral with regard to jurisdictional claims in published maps and institutional affiliations.

Author details

[1]Beijing Hospital of Traditional Chinese Medicine affiliated with Capital Medical University, No. 23, Back Road of Art Gallery, Dong Cheng District, Beijing 100010, China. [2]Beijing University of Chinese Medicine, No. 11, Bei San Huan Dong Lu, Chaoyang District, Beijing 100029, China.

Received: 7 November 2016 Accepted: 5 April 2017
Published online: 20 April 2017

References

1. National Comprehensive Cancer Network (NCCN). NCCN Clinical Practice Guidelines in Oncology: antiemesis. Version 1. Fort Washington: NCCN; 2013.
2. Chan VT, Yeo W. Antiemetic therapy options for chemotherapy-induced nausea and vomiting in breast cancer patients. Breast Cancer (Dove Med Press). 2011;3:151–60.
3. Inrhaoun H, Kullmann T, Elghissassi I, Mrabti H, Errihani H. Treatment of chemotherapy-induced nausea and vomiting. J Gastrointest Cancer. 2012;43(4):541–6.
4. Roila F, Hesketh PJ, Herrstedt J. Prevention of chemotherapy- and radiotherapy-induced emesis: results of the 2004 Perugia International Antiemetic Consensus Conference. Ann Oncol. 2006;17(1):20–8.
5. Hilarius DL, Kloeg PH, van der Wall E, van den Heuvel JJ, Gundy CM, Aaronson NK. Chemotherapy-induced nausea and vomiting in daily clinical practice: a community hospital-based study. Support Care Cancer. 2012;20(1):107–17.
6. Schnell FM. Chemotherapy-induced nausea and vomiting: the importance of acute antiemetic control. Oncologist. 2003;8(2):187–98.
7. Hawkins R, Grunberg S. Chemotherapy-induced nausea and vomiting: challenges and opportunities for improved patient outcomes. Clin J Oncol Nurs. 2009;13(1):54–64.
8. Navari RM. Management of chemotherapy-induced nausea and vomiting: focus on newer agents and new uses for older agents. Drugs. 2013;73(3):249–62.
9. Lin SJ, Hatoum HT, Buchner D, Cox D, Balu S. Impact of 5-HT_3 receptor antagonists on chemotherapy-induced nausea and vomiting: a retrospective cohort study. BMC Health Serv Res. 2012;12:215.
10. Grunberg SM. Obstacles to the implementation of antiemetic guidelines. J Natl Compr Canc Netw. 2009;7(5):601–5.
11. Salsman JM, Grunberg SM, Beaumont JL, Rogers M, Paul D, Clayman ML, et al. Communicating about chemotherapy-induced nausea and vomiting: a comparison of patient and provider perspectives. J Natl Compr Canc Netw. 2012;10(2):149–57.
12. Rithirangsriroj K, Manchana T, Akkayagorn L. Efficacy of acupuncture in prevention of delayed chemotherapy induced nausea and vomiting in gynecologic cancer patients. Gynecol Oncol. 2015;136(1):82–6.
13. Shen J, Wenger N, Glaspy J, Hays RD, Albert PS, Choi C, et al. Electroacupuncture for control of myeloablative chemotherapy-induced emesis: a randomized controlled trial. JAMA. 2000;284(21):2755–61.
14. Garcia MK, McQuade J, Haddad R, Patel S, Lee R, Yang P, et al. Systematic review of acupuncture in cancer care: a synthesis of the evidence. J Clin Oncol. 2013;31(7):952–60.
15. Yang X, Zhang S. A randomized control study on the clinical effects of ramosetron in prophylaxis of nausea and vomiting induced by cisplatin chemotherapy in patients with lung cancer [in Chinese]. Zhongguo Fei Ai Za Zhi. 2005;8(4):322–5.
16. Chan AW, Tetzlaff JM, Altman DG, Laupacis A, Gøtzsche PC, Krleža-Jerić K, et al. SPIRIT 2013 statement: defining standard protocol items for clinical trials. Ann Intern Med. 2013;158(3):200–7.
17. Oken MM, Creech RH, Tormey DC, Horton J, Davis TE, McFadden ET, et al. Toxicity and response criteria of the Eastern Cooperative Oncology Group. Am J Clin Oncol. 1982;5(6):649–55.
18. Zigmond AS, Snaith RP. The Hospital Anxiety and Depression Scale. Acta Psychiatr Scand. 1983;67(6):361–70.
19. Wilson MM, Thomas DR, Rubenstein LZ, Chibnall JT, Anderson S, Baxi A, et al. Appetite assessment: simple appetite questionnaire predicts weight loss in community-dwelling adults and nursing home residents. Am J Clin Nutr. 2005;82(5):1074–81.

Submit your next manuscript to BioMed Central and we will help you at every step:

- We accept pre-submission inquiries
- Our selector tool helps you to find the most relevant journal
- We provide round the clock customer support
- Convenient online submission
- Thorough peer review
- Inclusion in PubMed and all major indexing services
- Maximum visibility for your research

Submit your manuscript at www.biomedcentral.com/submit

BioMed Central
