# Supplementary material for: Efficacy of acupuncture in the prevention and treatment of chemotherapy-induced nausea and vomiting in patients with advanced cancer: a multi-center, single-blind, randomized, sham-controlled clinical research
Source: Chin Med. 2020 Jun 3;15:57. doi: 10.1186/s13020-020-00333-x (PMC7268447; doi:10.1186/s13020-020-00333-x)
Supplement: Supplementary file 2 — Additional file 2. Acupuncture therapy regimens and supplementary results. [file 13020_2020_333_MOESM2_ESM.docx]

Additional file 2

| Table S1. Acupuncture points and needles Manipulation in true acupuncture group. | |
| --- | --- |
| Acupuncture points | Insertion and Manipulation |
| RN12 (Zhongwan) | Perpendicular needling for 2.5–3.5 cm with reducing twirling |
| LR13(Zhangmen, bilaterally) | Oblique or perpendicular needling for 1.3–2.5 cm with reducing twirling |
| RN6 (Qihai) | Perpendicular needling for 2.5–3.5 cm with tonic twirling |
| ST25 (Tianshu, bilaterally) | Perpendicular needling for 2.5–3.5 cm with reducing twirling |
| PC6 (Neiguan, bilaterally) | Perpendicular needling for 1.3–2.5 cm with reducing twirling |
| ST36 (Zusanli, bilaterally) | Perpendicular needling for 2.5–3.5 cm with tonic twirling and electrical stimulation |

| Table S2. Results for HADS assessment | | | | | | |
| --- | --- | --- | --- | --- | --- | --- |
|  | True Acupuncture Group (n=62) | |  | Sham Acupuncture Group (n=58) | | *P* value |
|  | Mean (SD) | 95% CI |  | Mean (SD) | 95% CI |  |
| **Anxiety** |  |  |  |  |  |  |
| Baseline | 4.290(3.821) | 3.320~5.261 |  | 3.534(3.186) | 2.697~4.372 | 0.241 |
| Day 3 | 3.855(3.308) | 3.015~4.695 |  | 3.983(3.353) | 3.101~4.864 | 0.834 |
| Day 7(±1) | 3.645(3.393) | 2.784~4.507 |  | 3.707(3.346) | 2.827~4.587 | 0.920 |
| **Depress** |  |  |  |  |  |  |
| Baseline | 5.210(3.955) | 4.205~6.214 |  | 4.569(3.377) | 3.681~5.457 | 0.343 |
| Day 3 | 5.194(3.883) | 4.207~6.180 |  | 5.034(4.117) | 3.952~6.117 | 0.828 |
| Day 7(±1) | 4.581(3.660) | 3.651~5.510 |  | 5.034(3.893) | 4.011~6.058 | 0.512 |

Abbreviations: HADS: Hospital Anxiety and Depression scale; SD, standard deviation; CI, confidence interval.

*P values are based on independent-samples t tests comparing differences in between-group means.

| Table S3. Safety assessments | | | | |
| --- | --- | --- | --- | --- |
|  | True acupuncture group (n=65) | | Sham acupuncture group(n=60) | |
|  | Grade1 | Grade2-3 | Grade1 | Grade2-3 |
| **Adverse Events** |  |  |  |  |
| Diarrhoea | 0 | 0 | 0 | 2 |
| Constipation | 0 | 0 | 2 | 0 |
| Headache | 3 | 1 | 2 | 2 |
| **Laboratory tests** |  |  |  |  |
| ALT increased | 0 | 0 | 1 | 0 |
| Urea nitrogen increased | 1 | 0 | 0 | 0 |
| Creatinine increased | 1 | 0 | 0 | 0 |
